# *Bartonella* spp. Transmission by Ticks Not Established

**DOI:** 10.3201/eid1603.090443

**Published:** 2010-03

**Authors:** Sam R. Telford, Gary P. Wormser

**Affiliations:** Tufts University Cummings School of Veterinary Medicine, North Grafton, Massachussetts, USA (S.R. Telford III); New York Medical College, Valhalla, New York, USA (G.P. Wormser)

**Keywords:** Bartonella, ticks, Lyme disease, bacteria, parasites, perspective

## Abstract

*Bartonella* spp. infect humans and many animal species. Mainly because PCR studies have demonstrated *Bartonella* DNA in ticks, some healthcare providers believe that these microorganisms are transmitted by ticks. *B. henselae,* in particular, is regarded as being present in and transmissible by the *Ixodes scapularis* tick. The presence of a microbial agent within a tick, however, does not imply that the tick might transmit it during the course of blood feeding and does not confer epidemiologic importance. After a critical review of the evidence for and against tick transmission, we conclude that transmission of any *Bartonella* spp. by ticks, to animals or humans, has not been established. We are unaware of any well-documented case of *B. henselae* transmission by *I. scapularis* ticks.

## Abstract 2

A review found no well-documented case of transmission by deer ticks.

Infections with *Bartonella* spp. appear to be widespread in many animal species besides cats (*1*). Some evidence has been advanced in support of the possibility of tick transmission. Such findings have resulted in diagnostic testing and empiric therapies directed at *B. henselae* infection that are of dubious value with respect to illnesses thought to be caused by deer tick exposure. We critically examined the reported findings regarding tick transmission of *Bartonella* spp.

*Bartonella* spp. are common bacterial hemoparasites of mammals; for as long as 100 years, 2 species have been known to cause infections of public health significance. Trench fever, caused by *B. quintana* (formerly *Rochalimaea* *quintana*) and transmitted by body lice, affected hundreds of thousands of soldiers or displaced persons during World War I and to this day affects homeless persons. Oroya fever (and its chronic manifestation verruga peruana), caused by infection with *B. bacilliformis* and transmitted by phlebotomine sandflies, is a potentially severe febrile disease. Although it is geographically restricted to the high altitudes of the Andes and affects only a relatively small number of persons, the high case-fatality rate brought attention to this apparent anthroponosis as early as the late 1800s.

*B. henselae* causes cat-scratch disease, the most common *Bartonella* spp. infection in the United States (*2*). The hallmark of cat-scratch disease is enlargement and tenderness of lymph nodes draining the site of inoculation of the microorganism (*3*). In addition, a skin or mucous membrane lesion may be observed at the site of inoculation for 25% to >90% of patients (*3*,*4*). Extranodal clinical manifestations (e.g., encephalopathy, neuroretinitis, arthritis, and lytic bone lesions) occur in ≈10% of patients (*3*–*6*). Cats are the main reservoir of *B. henselae*. In a study from San Francisco, 25 (41%) of 61 pet, pound, or stray cats (*Felis domesticus*) were found to have *B. henselae* bacteremia (*7*). Bites or scratches from infected cats are associated with development of cat-scratch disease. The gut of cat fleas is commonly infected, and exposure to feces of infected fleas is the presumed route of transmission to uninfected cats and a possible route of transmission to humans.

Parasitologists focusing on blood parasites have long noted the ubiquity of *Bartonella* spp. within mammals, particularly rodents, and by the late 1960s nearly 2 dozen species had been described within the genus *Grahamella* (*8*). The genera *Rochalimaea* and *Grahamella* were subsumed into the genus *Bartonella* (*9*), and many of the validly published *Grahamella* spp. have been excluded from the list of approved bacterial taxa (*10*). These actions tended to foster ignorance of the history of the diversity of *Bartonella* spp. and to promote a fallacy in pathogen discovery (*11*); namely, if a DNA sequence is not present in GenBank, surely it must represent something novel, the extensive classical literature on a likely identical organism known only by morphology notwithstanding. The significance of such a fallacy is that a large body of literature that may provide critical details on the biology of a “novel” agent is completely overlooked or dismissed.

## Vector Relationships

Seminal studies by Richard Pearson Strong and the members of the American Red Cross trench fever commission (*12*) conclusively demonstrated biological as opposed to mechanical transmission of the trench fever agent by body lice. Feeding experiments on human volunteers established that lice may transmit by bite or by fecal contamination of abraded skin; that an infected louse remains infectious for at least 2 weeks; that the agent is not inherited by the progeny of infected lice; and that transmission may be extremely efficient, causing trench fever in 75% of volunteers after 1 exposure to a feeding box containing ≈50 lice that had previously fed on patients with trench fever.

Although initially Oroya fever was epidemiologically associated with ticks (*13*), it rapidly became evident that phlebotomine sandflies (particularly *Lutzomyia verrucarum*) were the vectors. Sandflies were the only blood-feeding arthropods that were peridomestic in their habits and occurred in the “bartonella zone,” >2,000 m elevation. Experimentally, sandflies acquired infection from blood-smear positive patients and transmitted infection by bite to those without evidence of *Bartonella* spp. infection (*14*).

Grahamellae (now bartonellae) of rodents have long been known to be transmitted by fleas (*15*–*17*). Such studies have noted the difficulty with which experimental infections may be established by means other than inoculation of flea homogenates, the persistence within the rectal sac of the flea, and the likely mode of perpetuation of the bacteria by larval fleas ingesting dried infected blood. In addition, grahamellae-infected rodents were noted to exist in the absence of ticks, demonstrating that ticks were not required to perpetuate these particular bacteria.

## Ticks as Vectors

Ticks are notorious vectors of a variety of agents that cause zoonotic infections (*11*), including viruses, bacteria, and protozoans. Like all animals, ticks have a diverse microflora. Recent analyses, using cloning and sequencing broad-range 16S rDNA amplification products, have documented a large bacterial flora within northeastern populations of *Ixodes scapularis* ticks that bite humans as nymphs, hereafter referred to as deer ticks (*18*,*19*). Amebas, mycoplasma, fungi, and helminths have been detected in these ticks by microscopy or other standard methods. However, the presence of a microbial agent within a tick does not imply that the tick might transmit it during the course of blood feeding or that it is pathogenic.

During early investigations of the causes of Oroya fever, Noguchi (*20*) demonstrated that *B*. *bacilliformis* could be experimentally transmitted between monkeys by the bites of *Dermacentor andersoni* ticks. However, the ticks that had been fed for a few days on infected monkeys were removed and allowed to reattach and complete their blood meal on uninfected animals, which became infected. Noguchi concluded that mechanical transmission had been demonstrated (perhaps by contamination of mouthparts or by regurgitation of the infectious partial blood meal), but persistence of viable bacteria or transstadial passage had not, and thus ticks were not biologic vectors.

Based on the volume of studies, the most compelling argument in favor of a tick vector for *Bartonella* spp. is that these microorganisms are sometimes detected in field-collected ticks (Table 1) (*15*). Although at least 20 studies have provided evidence for the presence of *Bartonella* spp. in primarily *Ixodes* spp. ticks collected at various locations in the United States and Europe, only 1 study has confirmed the presence of *Bartonella* spp. by culture (*15*,*21*,*22*). Caution is warranted when interpreting such data, however, because acquisition of *Bartonella* spp. from animal sources through a blood meal would be anticipated given the ubiquity of the microorganism in domestic animals and wildlife. In New England, as many as 60% of white-footed mice are blood-smear positive for *Grahamella* spp. (now *Bartonella*), regardless of collection site, including those trapped within the house of 1 of the authors where a tick life cycle was not present (S.R. Telford III, unpub. data); prevalence would probably reach unity if more sensitive modes of detection were used. The mere presence of *Bartonella* spp. or their DNA in ticks does not prove vector competence or confer epidemiologic significance (*15*), but it should serve as the impetus to rigorously perform the studies necessary to establish vector competence of ticks. At the least, viability should be established for bartonellae detected within ticks by means of in vitro cultivation.

**Table 1:** Reasons that *Bartonella* species might be transmitted by ticks

• Certain other arthropods can transmit *Bartonella* species.
• Seropositivity to *B. vinsonii* subsp. *berkhoffii* in dogs correlates with tick exposure and with seropositivity to other tick-borne pathogens. Seropositivity to *B. henselae* in feral cats in the United Kingdom correlated with seropositivity to *Borrelia burgdorferi*.
• *Bartonella* spp. DNA is present in ticks.
• Cases of *B. henselae* infection with preceding tick bite have been reported.
• Transstadial transmission of *B. henselae* in *Ixodes ricinus* ticks and transmission by *I. ricinus* ticks during a blood meal using an artificial feeding system have been shown.
• Case control study of cat-scratch disease found a significant association with having had a tick on the body, but this association lost statistical significance on a bivariate analysis controlling for kitten exposure.
• *Bartonella* spp. are commonly present in *Peromyscus leucopus* mice, a major host for deer ticks and a main reservoir of *Borrelia burgdorferi*.

To date, no report has documented transmission of *B. henselae* or any other *Bartonella* spp. to an animal after a tick bite (Table 2). The strongest evidence that ticks might be competent vectors for bartonellae was reported in a recent study in which *I. ricinus* ticks were infected with *B. henselae* in spiked (artificially infected) ovine blood by using an artificial feeding system (*23*). The ticks maintained infection throughout the molt, thereby establishing transstadial transmission. The experimentally infected ticks were also able to transmit *B. henselae* during a subsequent blood meal, again through the artificial feeding system; the dissected salivary glands from such ticks, when introduced into a cat, produced typical *B. henselae* infection, proving viability. Serious questions exist, however, as to whether these experiments are relevant to establishing vector competence. The ticks were fed continuously on blood meals with 10^9^ CFU/mL, representing a bacteremia that would rarely be seen in natural infections of cats. Given that *Ixodes* spp. nymphs ingest a total of ≈15 μL blood (*24*), each nymph may have ingested 10^6^–10^7^ bacteria, a large dose. In addition, the Houston-1 strain of *B. henselae* used in this study may not represent strains found in nature. It is highly adapted to the laboratory and readily grows in vitro, whereas primary isolates are extremely fastidious and grow slowly.

**Table 2:** Reasons that transmission of *Bartonella henselae* by deer ticks is unlikely or unproven

• Typical cat-scratch disease after a recognized deer tick bite has not been observed.
• Cat-scratch disease has a different seasonal pattern from that of Lyme disease.
• Appropriate seroepidemiologic studies have not been done.
• Vector competence of ticks for *B. henselae* in an animal system has not been proven.
• No convincing evidence of *B. henselae* in deer ticks has been reported.
• The *Bartonella* species present in *Peromyscus leucopus* mice is not *B. henselae*.
• The US cases with convincing evidence of *B. henselae* infection after a tick bite occurred in areas where Lyme disease is not endemic.

A more straightforward experiment to establish vector competence would be to feed an uninfected *Ixodes* sp. tick on a *B. henselae*–infected cat and then, after the tick has molted, determine whether *B. henselae* can be transmitted by tick bite to an uninfected cat. However, even if such an experiment were to prove vector competence, additional data would be needed to conclude that *Ixodes* spp. ticks are epidemiologically relevant as *B. henselae* vectors*.*

Do epidemiologic data that support tick transmission of *Bartonella* spp. in animals exist? One study correlated canine seropositivity to *B. vinsonii* subsp. *berkhoffii* with tick exposure and with seropositivity to other tick-borne pathogens (*25*). However, the dogs in that study were also heavily exposed to fleas, and according to findings with cats, flea transmission is as likely a possibility as tick transmission in dogs, if not more so (*15*,*25*,*26*). A study in the United Kingdom reported an association between seropositivity to *B. henselae* and to *Borrelia burgdorferi* in feral cats (*27*). The method used to detect antibodies to *B. burgdorferi* was not precisely described. However, the fact that the rate of seropositivity to *B. henselae* was nearly the same for domestic and feral cats, despite domestic cats having much less tick exposure than feral cats, raises questions about the epidemiologic relevance of tick transmission. In another study, a “novel” *Bartonella* subspecies was detected more often in white-footed mice concurrently infected with the tick-borne pathogens *B. burgdorferi* or *Babesia microti* (*1*), but this analysis failed to compare the likelihood that the *Bartonella* spp. might also commonly co-occur with rodent trypanosomes, which are maintained by fleas. Epidemiologic arguments must carefully control for confounding, and none to date argues convincingly for tick transmission of *Bartonella* spp.

## Studies of Humans

Certain authors have interpreted their studies as providing epidemiologic support for tick transmission of *Bartonella* spp. These data are, however, largely anecdotal and inconclusive (*28*,*29*). Culture-confirmed *B. henselae* infection was reported in 3 US patients who had been bitten by a tick within a few weeks of onset of illness (*28*,*30*); 2 of these patients had been in contact with a cat and may have been infected by this animal or its fleas. The tick species causing the bites was not identified for any of the patients but was unlikely to have been deer ticks because of the locations (Arkansas, Oklahoma, and probably North Carolina) (*30*), in which deer tick bites would be rare. *Bartonella* spp. have rarely (2 of ≈500 ticks) been detected in *Amblyomma americanum* ticks, the most common tick species to parasitize humans in these 3 states (*22*), but the finding was based on 1 PCR reaction and not confirmed with a second target or any other assay.

A more recent study described 3 patients from Europe for whom a scalp eschar and neck lymphadenopathy were attributed to tick transmission of *B. henselae* (*31*). Molecular detection of the microorganism by PCR of a biopsy specimen from the eschar, in conjunction with a high serum antibody titer by immunofluorescence assay, document *B. henselae* infection for 2 of the patients; a tick bite at the lesion site was presumed but not proven for either patient. Both had been in contact with cats that may well have transmitted this infection because the clinical features were indistinguishable from those of cat-scratch disease. The third patient, who had no cat exposure, had a documented bite from a *Dermacentor marginatus* tick that had PCR evidence of *B. henselae* infection. Whether the patient actually had *B. henselae* infection is questionable because PCR testing of tissue from the eschar was negative and antibodies to *B. henselae* could not be detected by immunofluorescence assay. The sole stated basis for the diagnosis was a positive Western blot result, but neither the interpretive criteria used nor the specificity of this testing were provided. When associated with a documented tick bite, the most common cause for a scalp eschar and neck lymphadenopathy is *Rickettsia slovaca*, but other rickettsia and even *Francisella tularensis* are possible causes, and in at least 25% of cases no pathogen can be identified (*31*).

Univariate analysis in a case–control study of cat-scratch disease in Connecticut found a significant association between having found a tick on the body and cat-scratch disease (*32*). This association, however, did not remain significant on multivariate (bivariate) analysis after controlling for exposure to kittens.

A 2001 report from New Jersey described 3 patients believed to have nervous system co-infection with *B. henselae* and *B. burgdorferi* (*33*). The authors suggested that bartonellae were transmitted by infected deer ticks because of the co-infection with *B. burgdorferi* and because the investigators detected *B. henselae* in a deer tick found in the household of 1 of these co-infected patients and in several deer ticks found on the pet cat of a fourth patient believed to have only *B. henselae* infection. PCR detection of DNA of both *B. burgdorferi* and *B. henselae* in the cerebrospinal fluid of these patients was the primary basis for the diagnosis of co-infection. An accompanying editorial, however, raised concerns about the validity of the diagnosis of both neuroborreliosis and neurobartonellosis in these patients (*34*). The clinical features were atypical for either infection, and the laboratory test results in support of these infections showed inconsistencies. In addition, 2 of the 3 authors had a potential conflict of interest; they were associated with a commercial laboratory that stood to gain financially from laboratory testing for *B. henselae*. The PCRs used by these investigators and others need careful scrutiny. In a later publication (*35*), the authors of the original NJ report conceded that the primers that they had used to amplify *B. henselae* DNA were insufficiently specific to warrant the conclusion that *B. henselae* was detected. BLAST (www.ncbi.nlm.nih.gov/blast/Blast.cgi) analysis of their primer P12B demonstrates identity with mouse mitochondrial DNA; also, what might be amplified if the PCR reaction were not stringent enough (e.g., lower annealing temperature) is not clear. In addition, their primer P24E contains a large proportion of α-proteobacterial 3′ terminus 16S rDNA consensus sequence. Because the specificity of PCR testing depends on target selection and reaction conditions, molecular detection using current primer sets may identify yet-undescribed genera of environmental bacteria distinct from *Bartonella* spp. Future examination of field-collected ticks for *Bartonella* spp. DNA should use a minimum of 2 independent PCR targets, preferably those that include larger portions of phylogenetically informative genes; to demonstrate viability, *Bartonella* spp. cultures should be attempted from all DNA-positive ticks. The deer ticks were unlikely to have been actually infected with *B. henselae* unless one postulates that feral cats serve as common hosts to larval or nymphal deer ticks. Indeed, the relatively high prevalence of reported *Bartonella* spp. infection (*35*) suggests that these ticks feed on cats as frequently as they do on mice. Although cats certainly serve as hosts for deer ticks of all stages, their contribution to feeding these vectors relative to all other animals remains to be defined and is likely to be minimal compared with rodents or birds. Given how frequently deer ticks feed on mice, *B. vinsonii arupensis* (previously known as *Grahamella peromysci*), which was isolated from a febrile, encephalopathic patient as well as from a patient who died from endocarditis, should more commonly infect persons in Lyme disease–endemic sites. This agent, however, has not been detected in deer ticks in any survey to date. Nevertheless, that *B. henselae* infection is a potential deer tick-transmitted co-infection in patients with possible Lyme disease is still widely accepted by the “chronic Lyme disease” counterculture (i.e., those physicians, patients, and activists who believe that patients with unexplained subjective symptoms have chronic *B. burgdorferi* infection even in the absence of exposure to a disease-endemic area or credible laboratory evidence of infection) (*36*).

Anecdotal accounts of *B. henselae* co-infection with *B. burgdorferi* in patients have been reported from Poland (*37*), Russia (*29*), and North Carolina (*38*). The report from North Carolina relied solely on immunoglobulin (Ig) M seroreactivity to *B. burgdorferi* to support a diagnosis of neuroborreliosis (*38*). The relatively poor specificity of IgM serologic testing (*39*) and the fact that the case was from outside Lyme disease–endemic regions of the United States raise concerns about the validity of the diagnosis of *B. burgdorferi* infection in this patient.

A straightforward approach to address whether *B. henselae* is transmitted by deer ticks would be seroepidemiologic studies to compare the prevalence of *B. henselae* antibodies in patients with Lyme disease with those in appropriate control groups, but such studies have not been performed. A study in Slovenia found that only 1 of the 86 children in whom febrile illness developed after a tick bite had Lyme disease in conjunction with seroconversion for IgG antibodies to both *B. henselae* and *B. quintana* (*40*).

In the United States alone, >20,000 cases of Lyme disease and about the same number of cases of cat-scratch disease occur annually (*2*). Thus, co-infections may occur occasionally by chance alone, without cotransmission by a tick vector. If the bite of a deer tick is a common route for *B. henselae* transmission, the absence of reports of the typical lymph node findings of cat-scratch disease proximal to the bite site of this tick species seems puzzling. The seasonality of cat-scratch disease, in which most cases in temperate regions occur in autumn and early winter (when peak breeding of cat fleas and birth of kittens occur), provides further evidence against a major role for ticks in transmission of *B. henselae* (*32*).

## Conclusion

Tick transmission of any *Bartonella* spp. to either animals or humans has not been established. *B. henselae* in particular is unlikely to be transmitted by deer ticks, and, to our knowledge, no well-documented case of transmission by this tick species in humans or animals has been reported.
